# Circumference-Based Predictions of Body Fat Revisited: Preliminary Results From a US Marine Corps Body Composition Survey

**DOI:** 10.3389/fphys.2022.868627

**Published:** 2022-03-31

**Authors:** Adam W. Potter, William J. Tharion, Lucas D. Holden, Angie Pazmino, David P. Looney, Karl E. Friedl

**Affiliations:** ^1^ Thermal and Mountain Medicine Division, US Army Research Institute of Environmental Medicine, Natick, MA, United States; ^2^ Military Performance Division, US Army Research Institute of Environmental Medicine, Natick, MA, United States; ^3^ Oak Ridge Institute for Science and Education (ORISE), Oak Ridge, TN, United States; ^4^ Human Performance Branch, US Marine Corps Training and Education Command, Quantico, VA, United States; ^5^ US Army Research Institute of Environmental Medicine, Natick, MA, United States

**Keywords:** military, DXA, bioelectrical impedance, abdominal girth, fat free mass

## Abstract

**Purpose:** Body composition assessment methods are dependent on their underlying principles, and assumptions of each method may be affected by age and sex. This study compared an abdominal circumference-focused method of percent body fat estimation (AC %BF) to a criterion method of dual-energy x-ray absorptiometry (DXA), and a comparative assessment with bioelectrical impedance (BIA), in younger (≤30 years) and older (>age 30 years) physically fit (meeting/exceeding annual US Marine Corps fitness testing requirements) men and women.

**Methods:** Fit healthy US Marines (430 men, 179 women; 18–57 years) were assessed for body composition by DXA (iDXA, GE Lunar), anthropometry, and BIA (Quantum IV, RJL Systems).

**Results:** Compared to DXA %BF, male AC %BF underestimated for both ≤30 and >30 years age groups (bias, -2.6 ± 3.7 and -2.5 ± 3.7%); while female AC %BF overestimated for both ≤30 and >30 years age groups (2.3 ± 4.3 and 1.3 ± 4.8%). On an individual basis, lean men and women were overestimated and higher %BF individuals were underestimated. Predictions from BIA were more accurate and reflected less relationship to adiposity for each age and sex group (males: ≤30, 0.4 ± 3.2, >30 years, -0.5 ± 3.5; women: ≤30, 1.4 ± 3.1, >30 years, 0.0 ± 3.3). Total body water (hydration) and bone mineral content (BMC) as a proportion of fat-free mass (FFM) remained consistent across the age range; however, women had a higher proportion of %BMC/FFM than men. Older men and women (>age 30 years) were larger and carried more fat but had similar FFM compared to younger men and women.

**Conclusion:** The AC %BF provides a field expedient method for the US Marine Corps to classify individuals for obesity prevention, but does not provide research-grade quantitative body composition data.

## Introduction

Anthropometrically-based body fat (BF) predictive equations have been widely used in sports physiology and have also been used in military physical readiness standards (readiness is defined as immediately prepared to perform full military duties) (Wilmore and Behnke, 1969; Wilmore and Behnke, 1970; Jackson and Pollock, 1978; Jackson et al., 1980; Hodgdon, 1992; Jones et al., 1992). Early evaluations of bioelectrical impedance analysis (BIA) concluded that BIA did not provide advantages over anthropometric assessment, but subsequent improvements in the BIA equations and re-evaluation of the methodology have made this a practical method which is also used in field research (Hodgdon and Fitzgerald, 1987; Jackson et al., 1988; Segal et al., 1988; Sun et al., 2003). The methods were initially developed against the criterion method of hydrodensitometry, with two-compartment interpretations of body density (Wilmore and Behnke, 1969; Wilmore and Behnke, 1970; Hodgdon and Beckett, 1984a; Hodgdon and Beckett, 1984b; Segal et al., 1985; Lukaski et al., 1986; Hodgdon and Fitzgerald, 1987; Segal et al., 1988; Hodgdon, 1992). These practical methods are based on underlying assumptions that can affect the interpretation of the results and guide the appropriate application of the method. Using a relevant test population of fit healthy active duty US Marines, this paper evaluated the accuracy of current military circumference equations and bioelectrical impedance equations for men and women compared to dual-energy x-ray absorptiometry (DXA), with a special focus on how the equations perform for women and older (>age 30) men and women. The DXA criterion method provides a three compartment model of body composition, separating the bone component from estimation of fat and lean components of soft tissue (Mazess et al., 1990).

Active duty Marine Corps volunteers in this study provided an ideal group to evaluate body composition methodologies in non-obese fit and healthy individuals. The Marine Corps has led military physical readiness standards including the use of body composition and circumference-based equations (Wright and Wilmore, 1974; Wright et al., 1980; Wright et al., 1981). A basic tenet is that every Marine is first a basic rifleman, and this focused mission is supported by a unique culture of physical fitness. Marines are required to maintain a high level of fitness and military bearing (physique) throughout their careers, and for purposes of this study they provide a reasonable representation of male and female athletes including older age master class athletes.

In the 1970s, the Marines were the first to adopt enforceable body composition standards, developing their own set of circumference-based equations derived from the work of Behnke and Wilmore (Wilmore and Behnke, 1969; Wilmore and Behnke, 1970; Wright and Wilmore, 1974; Wright et al., 1980; Wright et al., 1981). The important distinction between body size and body composition formed the basis of the Marines’ modernized readiness standards (Behnke et al., 1942; Welham and Behnke, 1942; Friedl, 2005). In 1980, President Jimmy Carter directed the military services to review policies and standards of fitness for a sedentary post-Vietnam era military and this led to a Department of Defense directive to all military services to follow the example of the Marines and develop enforceable body fat standards using circumference-based methods (US Department of Defense, 1981a; US Department of Defense, 1981b). Eventually, all the services agreed to a common set of equations developed by James Hodgdon at the Navy Health Research Center. These were developed against a sample of male and female sailors and validated against a large sample of male and female soldiers from the 1984 Army Body Composition Study (Hodgdon and Beckett, 1984a; Hodgdon and Beckett, 1984b; Fitzgerald et al., 1986). The Hodgdon equations are an integral part of the US Department of Defense body composition standards today (Hodgdon and Friedl, 1999).

While the equations are empirically calibrated to a total body fat assessment, the results are biased according to the nature of the underlying assumptions and measurement sites. The circumference equations used by the US military permit a regional focus on abdominal fat which enhances the relevance of the method for applications involving health, physical performance, and fit appearance, and the abdominal circumference (AC) is the single strongest anthropometric predictor of body fat in men (Lean et al., 1995; Kuk et al., 2005). This intended bias of the predictive equation for men does not fully translate to the same associations of body circumferences in women, where fat distribution is more varied and less likely to favor an abdominal location. However, both the male and female equations use measurements from body fat sites with the greatest lability (abdomen and hips) in response to chronic exercise and nutrition habits (Fried and Kral, 1987; Rebuffé-Scrive et al., 1987; Baumgartner et al., 1990; Pi-Sunyer, 2004). Inclusion of these other sites or assessment of a true total body fat would be discriminatory against groups of individuals with fat deposition in other sites that are not readily modifiable and that are less related to military health, performance, or appearance goals (Friedl, 2005). Nevertheless, prediction of militarily relevant body fat distribution in women is far more difficult than it is for men where %BF prediction and the key goals of the standard all converge on umbilicus AC. An investigation of the fairness of body fat standards for women highlighted four very different predictive equations for women, yet high consistency in how %BF was assessed in men (US General Accounting Office, 1998). This led to the universal adoption of the current Hodgdon equations, but questions persist about the adequacy of circumference-based predictions of %BF for women (Vogel and Friedl, 1992; Van loan et al., 2001).

Navy research in the 1940s also developed the use of total body water measurement as a method to predict body composition (Pace and Rathbun, 1945). This became even more useful in body composition studies with the discovery that electrical resistance of the body was directly related to total body water, expressed as stature^2^/resistance (Segal et al., 1985; Kushner et al., 1990; Kushner et al., 1992). In the 1980s, Army and Navy studies investigated the use of body water-based BIA as an alternative to anthropometrically-based methods (Hodgdon and Fitzgerald, 1987; Segal et al., 1988). Conclusions at that time were that BIA did not provide any significant advantage over AC %BF and there were concerns about other factors influencing the measurement, especially hydration status (Jackson et al., 1988). Further improvements in the equations and standardization of the methodology provide a valid physicochemical assessment of total body fat which is relevant at least to epidemiological studies (Sun et al., 2003). Assumptions about the hydration of the fat-free mass (FFM) are based on desiccation studies in several rodent species that, on average, produced values of 73.2% (Pace and Rathbun, 1945). This has been confirmed as a good estimate of the hydration of FFM in humans although there is a reported variability with age and sex (Hewitt et al., 1993; Going et al., 1995). Equations developed empirically against four compartment model body composition data populations yielded separate validated male and female equations (Sun et al., 2003). These equations were used in this study to test against DXA predictions with the sample of fit and healthy Marines.

## Methods

### Participants

This study was approved by the US Army Medical Research and Development Command Institutional Review Board and by the US Marine Corps Institutional Review Board. All participants provided written informed consent and women were provided with a rapid pregnancy test to establish absence of detectable pregnancy. All measures were obtained with the participants wearing Marine Corps athletic shorts and t-shirt, without shoes. A convenience sample of 609 US Marines including 430 men and 179 women, ages 18–57, and composed of 67% non-Hispanic white, 16% Hispanic, 6% black, 4% Asian, 1% Native American, and 6% multiple race/ethnicity volunteered for this study. Volunteers all self-reported their own race/ethnicity. This sample included 256 individuals from a sub-study of second lieutenants in the USMC Basic School. All Marines are required to maintain physical fitness and are evaluated annually in a Physical Fitness Test (PFT). The study sampled 0.3% of the US Marine Corps from multiple locations around the continental United States in order to obtain a representative sample, while also over-recruiting women (women currently comprise approximately 7% of the Marine Corps). A *post hoc* assessment found each of the sample subgroups to be adequate for a confidence interval of 95% within ±6% margin of error.

### Study Design

Stature and body mass were obtained with calibrated stadiometer and electronic floor scale (Seca, Chino, CA). Body mass index was calculated from these two variables. Body circumference measurements were made in accordance with the methodology described in MCO 6110.3A CH-3 (US Marine Corps, 2021). Briefly, circumferences were measured with an approved USMC tape measure (MyoTape, AccuFitness LLC, Denver, CO) that holds the tape flat against the skin to measure neck and abdomen (at the level of the umbilicus) for men, and neck, waist (at the thinnest portion of the abdomen), and hips (at the greatest protrusion of the buttocks) for the women. Abdominal circumference based percent body fat (AC %BF) was calculated for males = 86.010 × log10 [abdomen – neck (in)] - 70.041 × log10 [height (in)] + 36.76 and females = 163.205 × log10 [abdomen – neck (in)] – 97.684 x log10 [height (in)] − 78.387 (Hodgdon and Beckett, 1984a; Hodgdon and Beckett, 1984b). Reproducibility of this estimate between experienced observers is ±1% body fat units (Hodgdon and Friedl, 1999).

Body composition was assessed by DXA (iDXA, GE Healthcare, Madison, WI) and data analysis relied on manufacturer supplied algorithms (Encore, version 13.5, Lunar Corp., Madison, WI) (Mazess et al., 1990; Toombs et al., 2012; Lukaski, 2017). At the end of the DXA scan each volunteer remained in a relaxed supine position, on a nonconductive pad overlaying the wooden DXA platform, and total body resistance was measured at 50 KHz between left hand and left foot (Quantum IV, RJL Systems, Clinton Township, MI) and total body water (TBW) and body fat (%BF BIA) were calculated using the equations of Sun et al. (Sun et al., 2003). Disposable electrodes were placed mid-wrist on a line bisecting the ulnar head and at the base of the middle finger and mid-ankle on a line bisecting the medial malleolus and at the base of the middle toe.

Computed values were generated for body mass index (BMI) (body mass/stature^2^), fat-free mass index (FFMI) (FFM/stature^2^), hydration of FFM (%TBW/FFM), and bone mineral contribution to FFM (%bone mineral content/FFM).

Measurements were all taken on the same day during a single visit (<1 h). Each measure was obtained sequentially in order, as: 1) height, 2) body mass, 3) anthropometric circumference measures, 4) DXA measures, and lastly 5) BIA was conducted while still lying supine on the non-conductive surface of the DXA.

### Statistical Analyses

Data was analyzed using SPSS Version 26 (IBM Corporation, Armonk, New York) with Bland-Altman (Bland and Altman, 1986; Bland and Altman, 1999) analyses comparing AC %BF and BIA %BF to DXA %BF for men and women, age ≤30 and >30. Proportional values of %TBW/FFM and %BMC/FFM were plotted against age and linear regression analyses were performed. An analysis of variance (ANOVA), Concordance Correlation Coefficient (CCC) (Lin, 1989; Lin, 2000; Mcbride, 2005), and Coefficient of Determination were used to test differences and levels of agreement between ages and methods of %BF assessment in comparison to values from the DXA. Additionally, a comparison of FFM across groups and between BIA and DXA was conducted. Repeated measures ANOVA was conducted along with Mauchly’s test of sphericity to assess relations among measured data. For men sphericity was assumed; while women sphericity was not assumed and therefore Greenhouse-Geisser correction was applied.

## Results

Table 1 outlines all of the main computations from this study, including age group descriptive statistics and calculated outcome values.

**TABLE 1:** Total sample characteristics.

	Men		Women	
Age group (y)	≤30	>30	≤30	>30
n	255	175	103	76
Stature (cm)	176.6 ± 6.4	177.0 ± 6.5	163.8 ± 6.1	164.1 ± 6.8
Body mass (kg)	80.7 ± 9.2	86.4 ± 9.6	66.0 ± 8.2	66.7 ± 9.0
BMI (kg/m^2^)	25.8 ± 2.4	27.7 ± 2.6	24.6 ± 2.4	24.7 ± 2.6
^a^ FFM_BIA_ (kg)	65.9 ± 7.2	68.1 ± 7.3	48.0 ± 4.9	47.7 ± 5.2
FFM_DXA_ (kg)	66.8 ± 7.7	67.9 ± 7.6	49.2 ± 5.9	47.8 ± 5.8
FFMI (kg/m^2^) (DXA)	21.4 ± 1.8	21.7 ± 2.0	18.3 ± 1.7	17.7 ± 1.6
^a^ TBW (L)	47.8 ± 5.4	49.3 ± 5.5	34.5 ± 3.8	34.2 ± 4.0
%TBW FFM	71.6 ± 3.0	72.6 ± 3.5	70.3 ± 3.0	71.6 ± 3.7
BMC (kg)	3.3 ± 0.4	3.3 ± 0.4	2.6 ± 0.3	2.6 ± 0.3
%BMC FFM	4.9 ± 0.4	4.9 ± 0.4	5.2 ± 0.4	5.4 ± 0.4

a Calculated from Sun et al. (2003).

**FIGURE 1:**
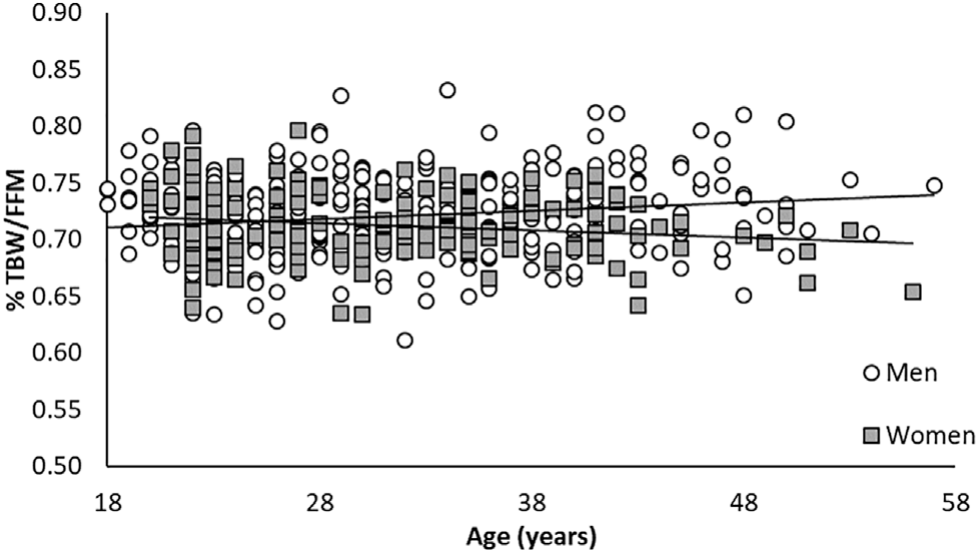
Relationship between %TBW/FFM across ages for men and women.

**FIGURE 2:**
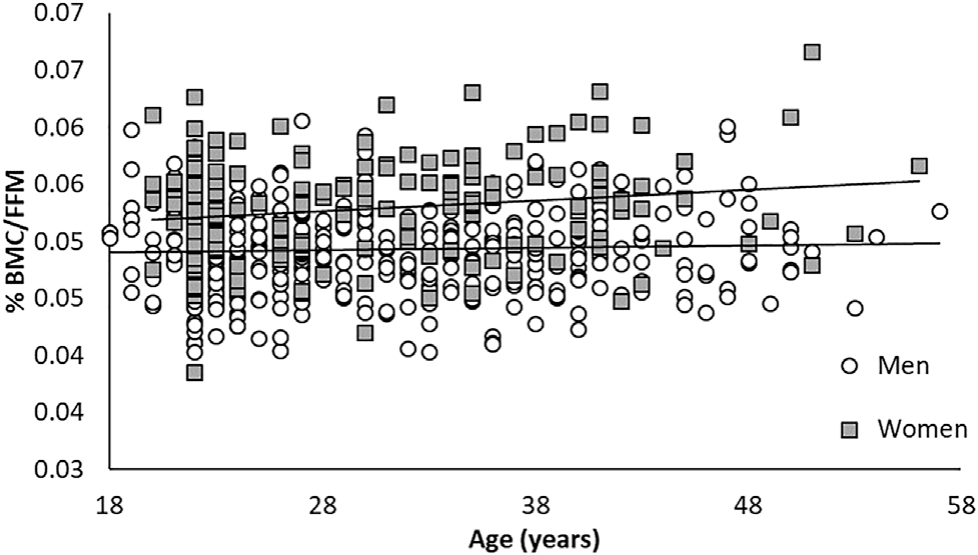
Relationship between %BMC/FFM across age for men and women.

Total body water (hydration) and bone mineral content (BMC) as a proportion of FFM remained similar between young and old. However, women had a higher proportional %BMC/FFM than the men (Figures 1, 2; Table 1). Older (>30 years) men and women were larger and carried more fat, but had similar FFM compared to younger men and women.

Within-subjects effects analyses for men between methods showed the AC %BF method was below the lower bounds of both BIA %BF and DXA %BF at the 95% confidence interval (CI); while the mean BIA %BF did not exceed the upper or lower of the 95% CI (Table 2). Analyses between age groups for men showed for ≤30 and >30 mean AC %BF was below the lower bounds of both BIA %BF and DXA %BF at 95%CI; while men ≤30 and >30 the mean BIA %BF did not exceed the upper bounds of DXA %BF at 95%CI (Table 3). Within-subjects effects analyses for women between methods showed the AC %BF method exceeded the upper bounds of DXA %BF at the 95% confidence interval (CI); while the mean BIA %BF did not exceed the upper or lower of the 95% CI (Table 2). Analyses between age groups for women showed for ≤30 mean AC %BF and BIA %BF exceeded the upper bounds of DXA %BF at 95%CI; while for women >30 the mean AC %BF and BIA %BF did not exceed the upper bounds of DXA %BF at 95%CI (Table 3).

**TABLE 2:** Within-subjects effects between %BF calculation methods.

Sex	Calculation method	Mean	Std. Error	95% confidence interval	
				Lower Bound	Upper Bound
Men	AC %BF	**17.25**	0.21	16.84	17.67
	BIA %BF	19.72	0.23	**19.27**	20.16
	DXA %BF	19.81	0.25	**19.32**	20.31
Women	AC %BF	**28.66**	0.39	27.89	29.43
	BIA %BF	27.58	0.38	26.83	**28.33**
	DXA %BF	26.88	0.44	26.02	**27.75**

Bold cells indicate mean values outside of BIA, and DXA %BF, lower bound reference values for men and bolded mean values outside BIA, and DXA %BF, upper bound for women.

**TABLE 3:** Within-subjects effects between %BF calculation methods and age groups.

Sex	Calculation method	Mean	Std. Error	95% confidence interval	
				Lower Bound	Upper Bound
Men ≤30	AC %BF	**15.11**	0.27	14.57	15.64
	BIA %BF	17.97	0.29	**17.40**	18.54
	DXA %BF	17.70	0.32	**17.07**	18.32
Men >30	AC %BF	**19.40**	0.33	18.76	20.04
	BIA %BF	21.46	0.35	**20.78**	22.15
	DXA %BF	21.93	0.39	**21.17**	22.69
Women ≤30	AC %BF	**27.89**	0.51	26.89	28.89
	BIA %BF	**27.03**	0.50	26.05	28.01
	DXA %BF	25.60	0.57	24.47	**26.72**
Women >30	AC %BF	29.43	0.59	28.27	30.60
	BIA %BF	28.13	0.58	26.99	29.27
	DXA %BF	28.17	0.66	26.86	29.48

Bold cells indicate mean values outside DXA %BF, lower and upper bound reference values.

**FIGURE 3:**
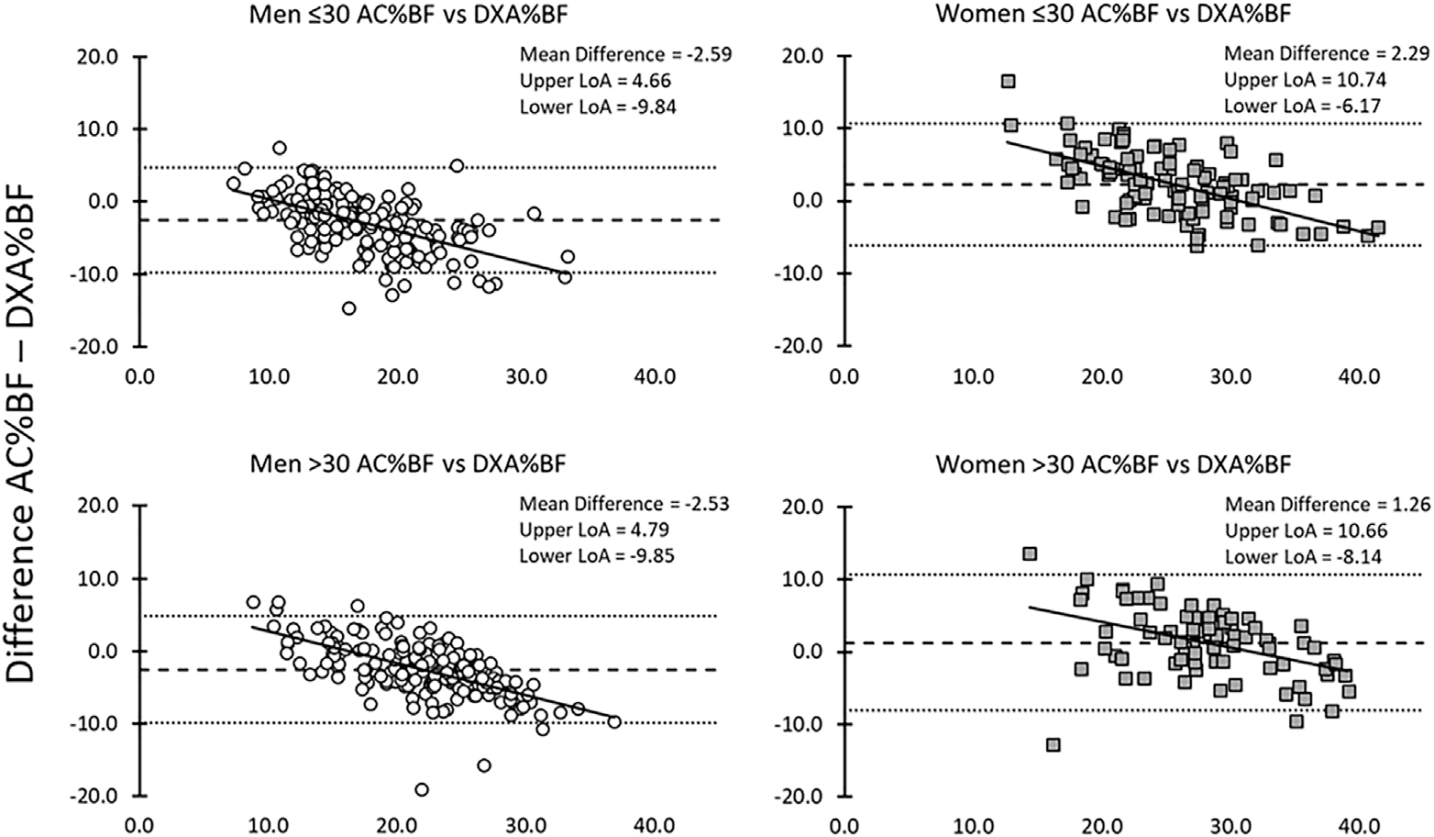
Bland-Altman AC to DXA %BF for men and women for each age group (≤30 and >30).

**FIGURE 4:**
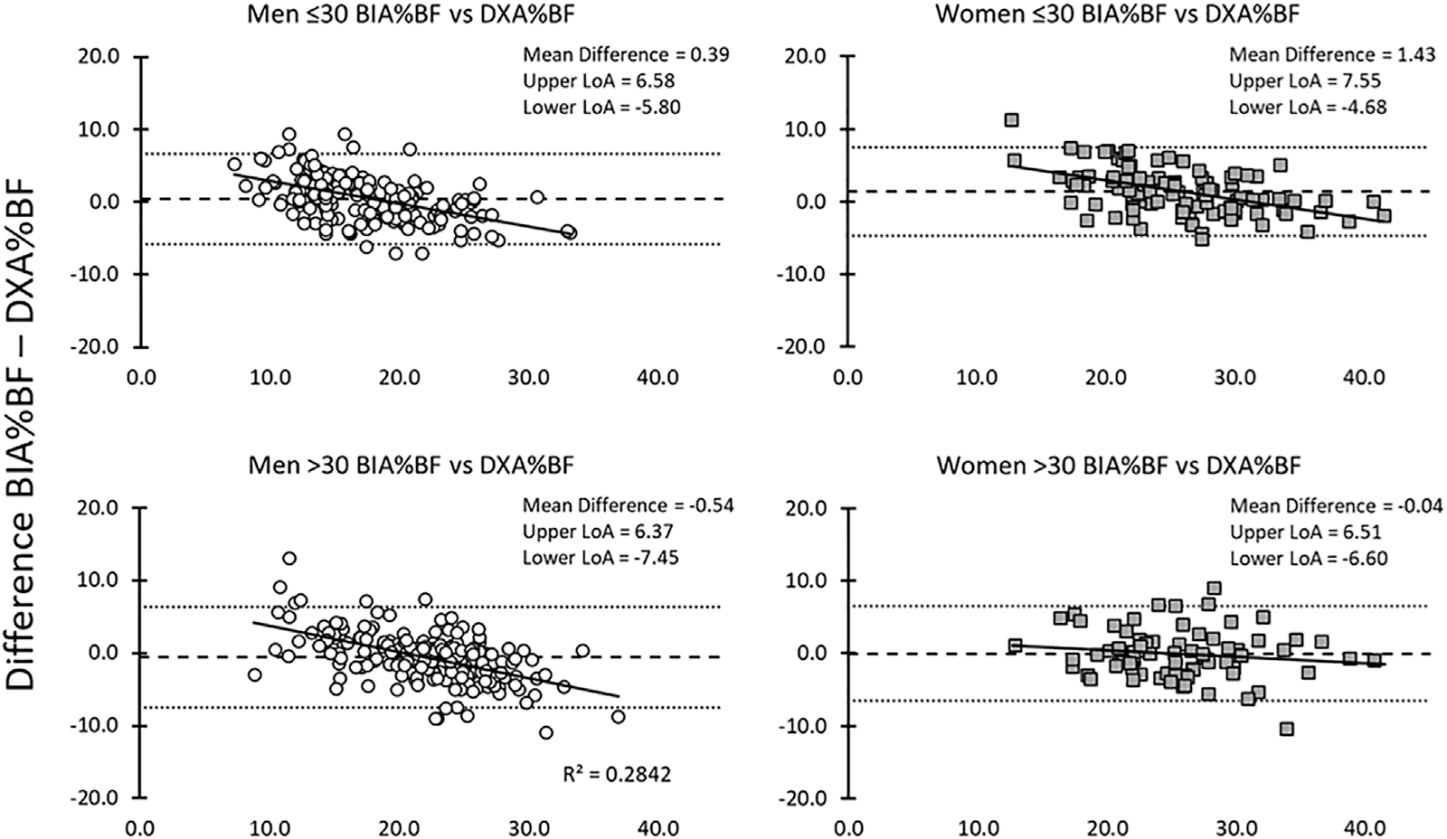
Bland-Altman BIA to DXA %BF for men and women for each age group (≤30 and >30).

Men %BF AC equations underestimated DXA%BF; while women overestimated %BF DXA. The largest overestimations occurred for the leanest women (Table 4; Figure 3). Predictions from %BF BIA were more accurate and reflected less relationship to adiposity (Table 4; Figure 4). Trend lines plotted to each age and sex group prediction show the error pattern in relationship to %BF (Figures 3, 4). Concordance Correlation Coefficient (CCC) calculations showed the AC %BF method and BIA %BF methods to have stronger correlation to the DXA %BF method for women over the men in both age groups and higher correlations in BIA %BF over AC %BF in all groups. Coefficient of determination calculations showed a higher amount of the variation between test measures (AC and BIA) could be explained based on actual difference in the criterion DXA measures (Table 4).

**TABLE 4:** Within-subjects effects between %BF calculation methods and age groups.

	Men		Women	
Age group (y)	≤30	>30	≤30	>30
%BF_DXA_	17.7 ± 5.0	21.9 ± 5.2	25.6 ± 5.7	28.2 ± 5.8
^a^ %BF_AC_	15.1 ± 4.4	19.4 ± 4.1	27.9 ± 4.7	29.4 ± 5.7
^b^ %BF_BIA_	18.1 ± 4.5	21.4 ± 4.4	27.0 ± 5.0	28.1 ± 5.0
Diff %BF AC-DXA	-2.6 ± 3.7	-2.5 ± 3.7	+2.3 ± 4.3	+1.3 ± 4.8
Diff %BF BIA-DXA	+0.4 ± 3.2	-0.5 ± 3.5	+1.4 ± 3.1	0.0 ± 3.3
CCC %BF AC:DXA	0.57	0.51	0.74	0.72
COD %BF AC:DXA	0.32	0.26	0.54	0.52
CCC %BF BIA:DXA	0.64	0.63	0.82	0.79
COD %BF BIA:DXA	0.41	0.39	0.68	0.63

DIFF, difference; CCC, concordance correlation coefficient; COD, coefficient of determination.

a calculated from Hodgdon and Beckett (1984a); Hodgdon and Beckett (1984b).

b calculated from Sun et al. (2003).

**FIGURE 5:**
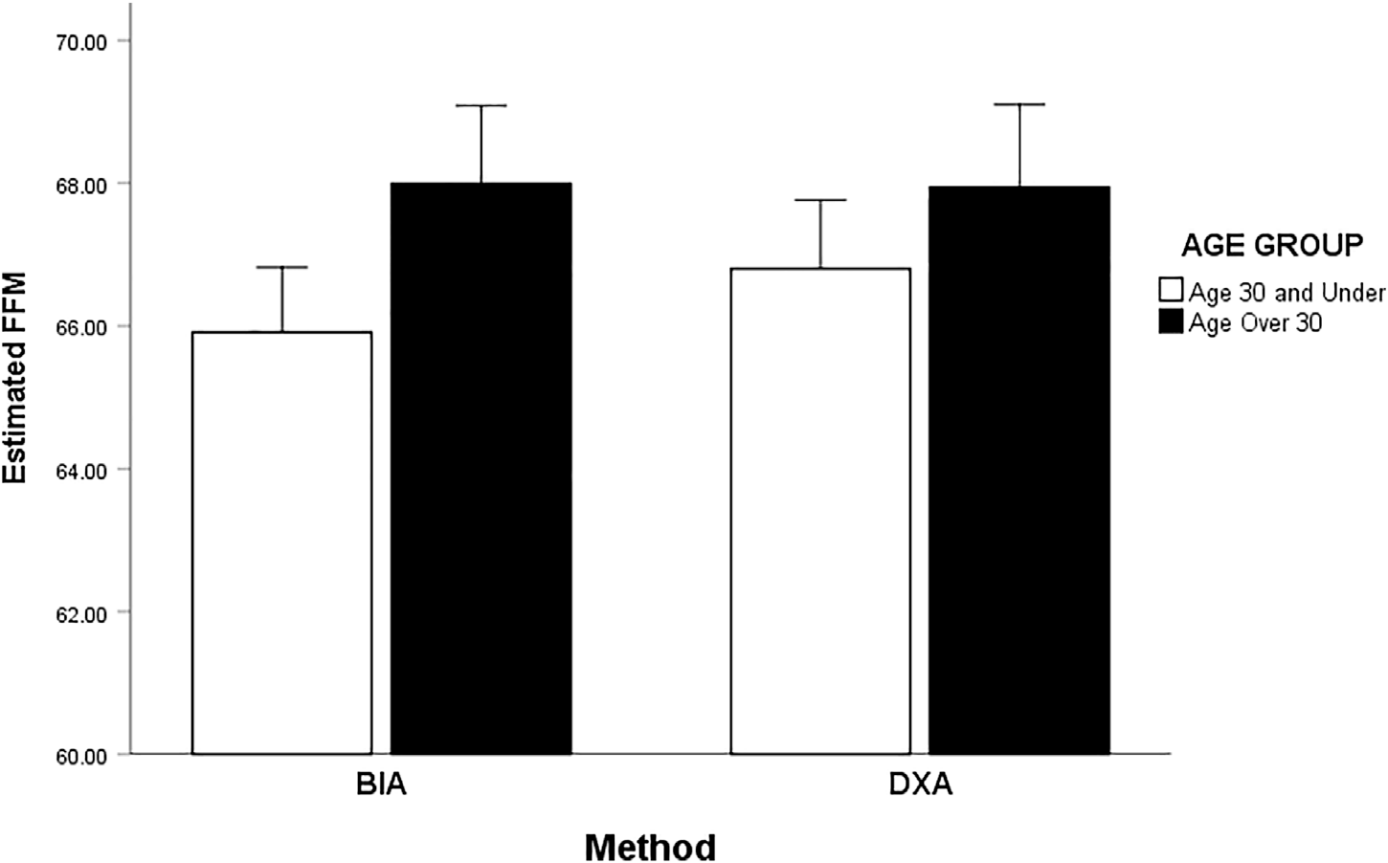
Interaction effect of FFM by age and method BIA and DXA for men (≤30 and >30).

**FIGURE 6:**
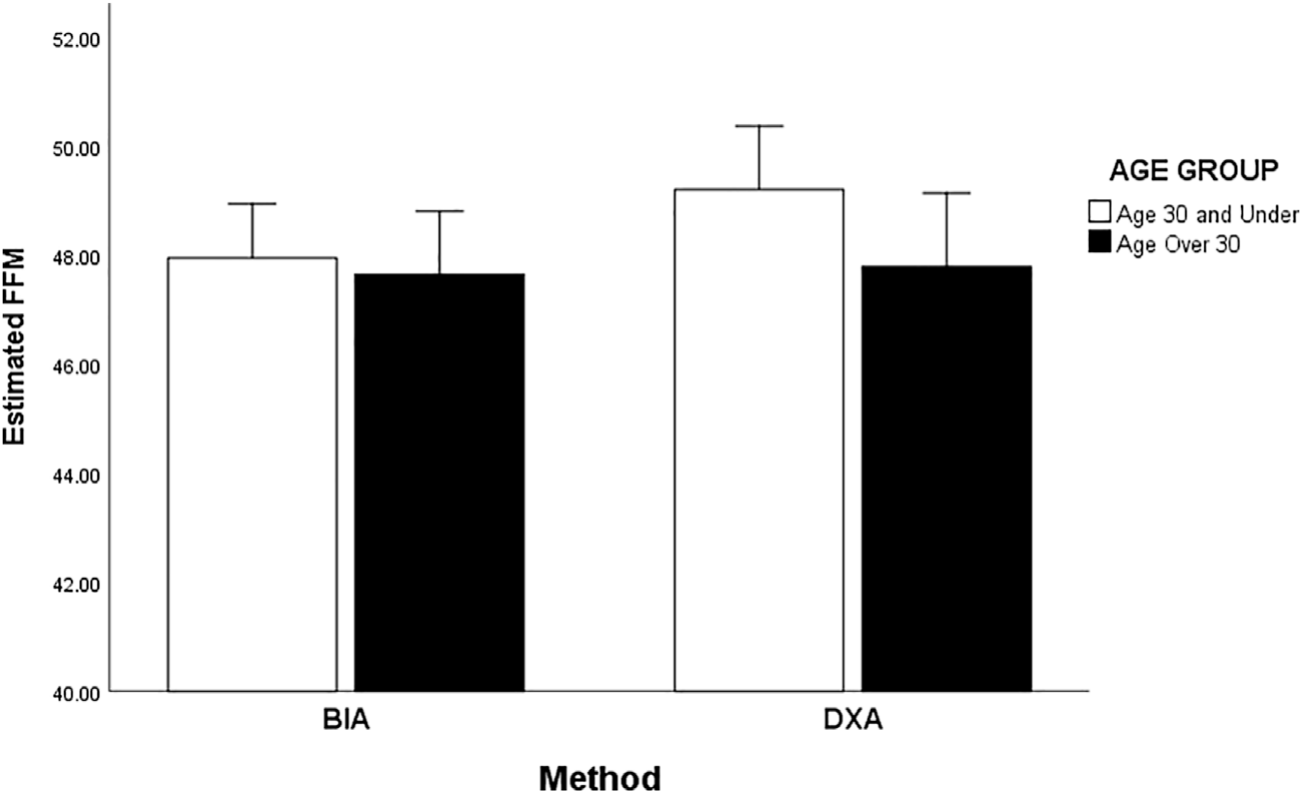
Interaction effect of FFM by age and method BIA and DXA for women (≤30 and >30).

Men ≤30 have significantly less FFM than Men >30 (Figure 5). However, those ≤30 show less FFM when measured by BIA compared to DXA whereas those over >30, assessment method has little effect on their FFM measure. Women ≤30 have significantly more FFM as assessed by DXA then women ≤30 assessed by BIA or women aged >30 assessed by either method. In comparison to DXA, BIA underestimates women’s FFM for those ≤30 but reasonably estimates those >30 (Figure 6). Women >30 have less FFM than women ≤30 but a main effect of age group is removed by the interaction of the method.

## Discussion

For this cohort of healthy fit Marines, AC %BF underestimated DXA %BF for men, but not for women, and age was not a major factor in the performance of the equations. The overestimation in women was influenced by the prevalence of relatively lean Marine participants, where AC %BF is known to overestimate lean men and women and tends to underestimate higher fat individuals. This trend is clearly reflected in the Bland-Altman plots (Figures 3, 4). At lower %BF, increasing amounts of intra-abdominal fat are relatively undetectable until abdominal girth measurements begin to reflect the increasing fat. For women, this generally starts at a higher level of fat accumulation than for men, as the female abdomen is generally protected against initial fat storage up to nearly ∼30 kg (Kvist et al., 1988). At the higher levels of %BF, the increased distribution of fat to other sites on the body (e.g., extremities and upper back) results in increasing underestimation of actual total %BF, with some increases in fat mass that are not captured in the lower trunk circumference measurements. The most accurate AC %BF predictions occur in the ranges bracketing current military body fat thresholds for men (18–26%) and women (26–36%), which has made this method suitable for proper categorization of individuals for %BF standards since the 1980s. However, the degree to which these equations still match to physique of the current generation of Marines is the subject of a larger study and analysis currently underway. These data make it clear that AC %BF is not suitable for applications requiring quantitative body composition assessment.

Compared to AC %BF, BIA %BF provide a more accurate reflection of DXA %BF, with closer measurements and smaller variance in the Bland-Altman plots (Figures 3, 4). However, like the anthropometric predictions, BIA %BF tended to overestimate %BF of lean men and women and underestimate %BF of men and women with the highest relative fat. FFM was higher in older men than younger, but higher in younger women than older; FFM was underestimated by BIA in younger women. The effect of overestimation at the lower end of body fat by bioelectrical impedance, as with predictions from anthropometry, was noted by Hodgdon and Fitzgerald in their analyses of BIA in earlier military studies (Hodgdon & Fitzgerald, 1987). This overestimation of %BF would also be expected if hydration of the FFM was lower than the normal assumptions of 72–74%. Although not measured with an independent method such as stable isotope water dilution, TBW calculated from resistance represented a smaller proportion of the DXA-assess FFM than the normal assumptions. There was no significant change across age for %TBW/FFM or %BMC/FFM in this cohort (Figures 1, 2). The influence of chronic exercise, including resistance exercise, on hydration of the FFM could be a factor and bears further investigation. We did not assess hydration status in this study, choosing to treat it as a real-life variation. In field studies using longitudinal testing with BIA, hydration status should be monitored with simultaneous urine specific gravity measurements and other expedient techniques such as estimation of acute blood volume change (Dill and Costill, 1974). Total body water has been used to further improve interpretation of density measurements in three compartment models, as defined in the 1959 body composition techniques summit meeting in Natick, Massachusetts (Brozek and Henschel, 1961). Electrical impedance has been used to obtain the TBW component for 3- and 4-compartment models, when greater accuracy is needed. As an example, BIA was used in the 4-compartment model criterion method for a study validating the female circumference equation across race and ethnicity (Van loan et al., 2001).

Anthropometrically defined physique may be more important in field studies than attempts to reflect physiochemically defined whole body composition. For example, in training studies it may be more useful to define exercise-induced changes by assessing body shape (physique) reflected by upper body, lower trunk (abdominal), arm (biceps), and leg (mid-thigh) circumferences. As highlighted in the introduction, these two methods (AC and BIA %BF) depend on underlying assumptions. While AC %BF relies on consistency of how excess fat is reflected in truncal circumferences; BIA %BF is dependent on a consistent hydration of the FFM. These considerations are important for exercise physiologists or other researchers to account for when selecting the method best suited to their training, monitoring, or research applications (Lukaski, 2017). In future work, it is also critical to take into account both physical and physiological sexual dimorphism between men and women. This is especially important given there are known differences in both total and regional fat distribution between the sexes. A much more detailed assessment of physique and sex differences might be accomplished with relatively portable 3D scanners as these become more affordable (Harty et al., 2020). Combined techniques such as BIA and 3D scan measurements are likely to be most useful for field studies in the near future.

The US Marine Corps is a culture of fitness, where individual fitness is a high priority, personal responsibility, and ultimately a requirement for advancement. As such, this population is highly representative of the healthy exercising American population but does not represent the physical fitness and body composition characteristics of the general public, where the mean DXA %BF is nearly 25 and 36% for men and women, aged 20-30, compared to 18 and 26%, respectively, for the <30 year old Marines; while older Marines were also leaner than the general American public, and all these Marines had higher mean lean mass and BMC than their civilian counterparts (Chumlea et al., 2002; Kelly et al., 2009). A limitation to these analyses is that results may have differed if the state of individual participants were more controlled in terms of time of day for testing, meal timing, and hydration status; while these factors were not controlled in this study with real world sampling.

## Conclusion

Ultimately, the primary use of these predictive equations in the DoD is focused on modifiable truncal region fat and total body fat by DXA or even BIA are imperfect criterion methods. The AC %BF method may be useful for standards categorization but is not valid as a quantitative research tool. The real test of these equations for classifying individuals for excess fat or insufficient muscle mass would be the strength of the association with physical readiness and physical fitness performance (Friedl, 1992; Friedl, 2017). US Marines in this sample were consistently lower in %BF, higher fat free mass index, and higher bone density than their civilian peers, representing a culture of fitness and long term health (Chumlea et al., 2002; Kelly et al., 2009). These results and the results from current ongoing studies will further evaluate body composition methodology that can best serve the goals of military physical readiness standards. The results from this fit and healthy population of Marines are relevant to fit and healthy athlete or other military populations.

## Data Availability

The raw data supporting the conclusion of this article will be made available by the authors, without undue reservation.
